# Probing Nanoelectroporation and Resealing of the Cell Membrane by the Entry of Ca^2+^ and Ba^2+^ Ions

**DOI:** 10.3390/ijms21093386

**Published:** 2020-05-11

**Authors:** Wenfei Bo, Mantas Silkunas, Uma Mangalanathan, Vitalij Novickij, Maura Casciola, Iurii Semenov, Shu Xiao, Olga N. Pakhomova, Andrei G. Pakhomov

**Affiliations:** 1 Frank Reidy Research Center for Bioelectrics, Old Dominion University, Norfolk, VA 23508, USA; 201411040603@std.uestc.edu.cn (W.B.); msilkuna@odu.edu (M.S.); umangala@odu.edu (U.M.); casciolamaura@gmail.com (M.C.); isemenov@odu.edu (I.S.); SXiao@odu.edu (S.X.); opakhomo@odu.edu (O.N.P.); 2 School of Electronic Science and Engineering, University of Electronic Science and Technology of China, Chengdu 610054, China; 3 Department of Biology, Vytautas Magnus University, 44248 Kaunas, Lithuania; 4 Institute for Digestive System Research, Lithuanian University of Health Sciences, 44307 Kaunas, Lithuania; 5 Faculty of Electronics, Vilnius Gediminas Technical University, 03227 Vilnius, Lithuania; vitalij.novickij@vgtu.lt; 6 Department of Electrical and Computer Engineering, Old Dominion University, Norfolk, VA 23508, USA

**Keywords:** electroporation, electropermeabilization, membrane repair, membrane integrity, nsPEF, nsEP, nanopores

## Abstract

The principal bioeffect of the nanosecond pulsed electric field (nsPEF) is a lasting cell membrane permeabilization, which is often attributed to the formation of nanometer-sized pores. Such pores may be too small for detection by the uptake of fluorescent dyes. We tested if Ca^2+^, Cd^2+^, Zn^2+^, and Ba^2+^ ions can be used as nanoporation markers. Time-lapse imaging was performed in CHO, BPAE, and HEK cells loaded with Fluo-4, Calbryte, or Fluo-8 dyes. Ca^2+^ and Ba^2+^ did not change fluorescence in intact cells, whereas their entry after nsPEF increased fluorescence within <1 ms. The threshold for one 300-ns pulse was at 1.5–2 kV/cm, much lower than >7 kV/cm for the formation of larger pores that admitted YO-PRO-1, TO-PRO-3, or propidium dye into the cells. Ba^2+^ entry caused a gradual emission rise, which reached a stable level in 2 min or, with more intense nsPEF, kept rising steadily for at least 30 min. Ca^2+^ entry could elicit calcium-induced calcium release (CICR) followed by Ca^2+^ removal from the cytosol, which markedly affected the time course, polarity, amplitude, and the dose-dependence of fluorescence change. Both Ca^2+^ and Ba^2+^ proved as sensitive nanoporation markers, with Ba^2+^ being more reliable for monitoring membrane damage and resealing.

## 1. Introduction

Permeabilization of the cell membrane by intense electric field pulses, or electroporation, has been known for decades and has found numerous applications in gene and drug delivery, cancer ablation, membrane biophysics research, as well as in food processing and decontamination [1,2,3,4,5]. Nowadays, electropermeabilization research has expanded to the nanosecond-range pulsed electric fields (nsPEFs), revealing new and different bioeffects. Specifically, nsPEFs are distinguished (1) by the ability to disrupt intracellular membranous structures, such as endoplasmic reticulum and mitochondria [6,7,8,9], (2) by the phenomenon of bipolar cancellation, which stands for the suppression of diverse bioeffects upon the nsPEF polarity reversal [10,11,12,13], and (3) by the predominant formation of smaller membrane defects, often referred to as “nanopores” or “nanoelectropores,” although the exact nature of these defects remains uncertain [4,9,14,15,16,17,18]. Nanopores may remain open for minutes, and demonstrate complex conductive properties unexpected from simple “holes” in a lipid bilayer, including inward rectification, current and voltage sensitivity, and ion selectivity [15,16]. Some of these properties are similar to those of funnel-shaped artificial nanopores in polymer films [19], but it is difficult to explain how any conical lipidic pores would form and persist, for a long time, in a cell membrane which is only a few nanometers thick. An alternative explanation of the complex conductive properties of nanopores is that they are endogenous ion channels or other membrane proteins damaged or altered by the strong electric field, or perhaps they are some unique structures in the membrane made of both proteins and lipids [15,16,20]. It is also possible that there are several different types of electropores, which contribute differently to the permeabilized membrane state.

The need to understand the nature of nanopore formation by nsPEF and their properties is emphasized by the fact that nanopores give rise to a wide range of downstream effects, from the activation of second messenger signaling, depolarization, and excitation, to cell swelling, blebbing, actin disassembly, loss of the mitochondrial membrane potential, and necrotic or apoptotic cell death. There are three principal techniques which enable the analysis of different nanopore features [15,21]:

(1) Whole-cell patch clamp, which is arguably the most sensitive and quantitative technique. It is, however, most laborious (one cell at a time) and prone to diverse artifacts due to the nsPEF pickup by the amplifier. It requires either a temporary disconnection of the amplifier when nsPEF is applied [22], or nsPEF exposure of cells prior to the whole-cell formation [17,23], or a confirmation of patch-clamp data by independent (e.g., optical) methods [11,15,16,24,25].
(2) Measurements of changes in the cell or bleb volume, which result from the colloid-osmotic water uptake or loss [14,15,26]. This method is surprisingly sensitive to the molecular size of the solute, which is admitted (or not) into the cell through nanopores, thereby yielding an accurate estimate of pore size. The measurements are, however, indirect and relatively slow.
(3) Fluorescence detection of the entry or leakage of ions or dyes is by far the most common method of detection and quantitation of membrane electropermeabilization [13,15,21,27,28,29,30]. Dyes like propidium (Pr) iodide and YO-PRO-1 chloride display little fluorescence outside of the cell and have poor or no permeability through the intact cell membrane. Once the membrane integrity is compromised, the dyes enter the cell and bind to nucleic acids, which greatly enhances the fluorescence signal. The method is fast and semi-quantitative, as the emission gain is proportional to the number of dye molecules entering the cell in a given time interval [21,30].

However, the size of the dye molecules (more precisely, the size of Pr and YO-PRO-1 cations) restricts their passage through membrane pores. YO-PRO-1 is smaller than Pr and is a better marker for nanopore detection, but there are still smaller pores which do not admit either dye. Earlier, we proposed to load cells with a Tl^+^-sensitive fluorescent dye and monitor Tl^+^ entry from the outside as a measure of nanopore transport [28,31]. This method is far more sensitive for nanopore detection than either YO-PRO-1 or Pr uptake; it compares in the sensitivity with the patch clamp but is devoid of any electrical pickup artifacts. The major drawback is that Tl^+^ precipitates in solutions with Cl^−^, which puts unusual restrictions on the formulation of extracellular media. Also, Tl^+^ can be admitted “spontaneously” into intact cells through constitutively open K^+^ channels (and perhaps some other channels), so the dye fluorescence gradually goes up and limits the time interval when the entry by nanopores can be reliably measured.

Using Ca^2+^ entry as a marker of nanoporation does not require special media, a variety of Ca^2+^- sensitive fluorophores are available, and the detection sensitivity is similar to Tl^+^ entry [8,24,26,29,31,32,33,34,35]. Cytosolic Ca^2+^ level is tightly controlled, hence, in quiescent cells, the dye fluorescence can be stable for a long time. However, Ca^2+^ entry through nanopores should be separated from the activation of cation channels which can admit Ca^2+^ [24,29,34,35,36], as well as from receptor- and store-operated Ca^2+^ entry [37]. Moreover, a modest Ca^2+^ entry through the electropermeabilized membrane may trigger an overwhelmingly strong response by activating calcium-induced calcium release (CICR), followed by massive active pumping of excess Ca^2+^ from the cytosol [33]. Cytosolic Ca^2+^ level depends on many Ca^2+^- and time-dependent processes [38], which can mask Ca^2+^ flow through nanopores and obstruct quantitative measurements.

The goal of this study was to test if other divalent cations, which, unlike Ca^2+^, are not involved in the intracellular signaling, may be detected with a Ca^2+^ sensitive fluorophore and serve as more quantitative markers of nanoporation. We found that Ba^2+^ entry, as recorded by time-lapse fluorescence imaging with Calbryte dye, has nsPEF sensitivity similar to that of Ca^2+^ uptake, and much better than either YO-PRO-1 or Pr uptake. At the same time, Ba^2+^ does not trigger CICR and accumulates in the cytosol, making it more suitable than Ca^2+^ for quantitative measurements.

## 2. Results

### 2.1. Intact Cell Permeability to Cd^2+^, Zn^2+^, Ba^2+^, and Ca^2+^

**Figure 1:**
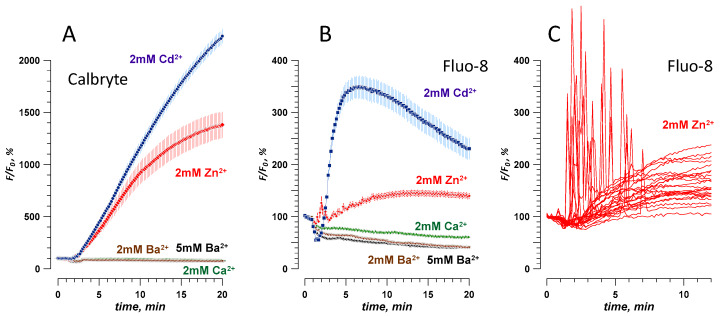
Effect of extracellular divalent cations on the fluorescence of CHO cells loaded with Calbryte (**A**) or Fluo-8 (**B**,**C**) dyes. The time-lapse imaging began in a physiological solution with 2 mM Ca^2+^ (considered as a baseline, 100%), which was replaced at 1–2 min by a modified solution with a divalent cation marked in the legend (see Methods for details). Note that 2 mM of Cd^2+^ or Zn^2+^ increased the emission of either dye, while Ca^2+^ and Ba^2+^ did not. Mean +/− s.e., *n* > 30 cells per group. Panel C shows fluorescence traces of the response to Zn^2+^ from several individual cells loaded with Fluo-8, without averaging.

To serve as a useful nanoporation marker, an ion should not elicit a dye signal in intact cells that have not been exposed to nsPEF. CHO cells, which typically do not express any endogenous voltage-gated channels [15,17,23,39], were loaded with a Ca^2+^-sensitive fluorophore Calbryte or Fluo-8. Time-lapse imaging of the dye fluorescence started when cells were bathed in a standard physiological solution with 2 mM Ca^2+^ (see Methods). In a few minutes, this solution was replaced with the same solution but containing 2 mM of Cd^2+^, Zn^2+^, or Ba^2+^. To control for possible mechanical disruption, we replaced the initial Ca^2+^-containing solution with the same Ca^2+^-containing solution. We found that switching to Cd^2+^ or Zn^2+^ solutions strongly increased the emission of both dyes (Figure 1A,B). The response to Cd^2+^ was a gradual increase (probably indicating Cd^2+^ entrance into the cell), whereas Zn^2+^ triggered a series of brief spikes, probably due to the mobilization of the intracellular Ca^2+^ (Figure 1C). Regardless of the exact mechanism of dye activation by Cd^2+^ and Zn^2+^ (which was beyond the scope of this study), these two ions were regarded unsuitable as nanoporation markers.

In contrast, dye emission did not change in Ba^2+^- or Ca^2+^-containing solutions, even when Ba^2+^ concentration was increased to 5 mM (Figure 1A,B). This result indicates that Ba^2+^ did not penetrate into intact cells “spontaneously,” which is consistent with its common use as a charge carrier in patch-clamp studies of Ca^2+^ channels [24,40,41]. Therefore, Ba^2+^ was chosen for the next experiments and compared to Ca^2+^ as a marker of electroporation. Although either Calbryte or Fluo-8 dye could be used, we chose to stay with Calbryte for its stronger and more consistent response.

### 2.2. Effect of a Single 300-ns Pulse at Different Electric Field Strengths

**Figure 2:**
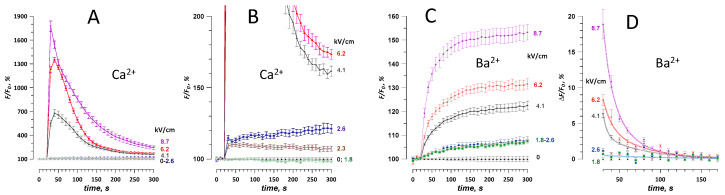
Traces of Ca^2+^ (**A**,**B**) and Ba^2+^ (**C**,**D**) fluorescence change in CHO cells in response to a single 300-ns pulse at the indicated electric field strength (kV/cm). Images were taken every 10 s, and the pulse was delivered at 27 s into the experiment. Panels A and B show the same data at different vertical scales, to emphasize the statistically significant response already at 2.3 kV/cm (*p* < 0.01 compared to 0 kV/cm, two-tailed *t*-test) and its disproportional enhancement by the calcium-induced calcium release (CICR) once its threshold is exceeded (see text and [33] for more detail). Panel D uses the data from panel C to plot the fluorescence change per 10-s intervals between the sequential readings. The values were fitted with a double-exponential function (see text and [11] for detail). Mean +/− s.e., *n* = 30–45 cells for most groups.

The nsPEF effects on Ca^2+^ activation in CHO cells have been studied in detail earlier, with FURA-2 and Calcium Green dyes [8,12,33,37,42]. Here, we observed a similar response using Calbryte dye, and compared it side by side to the nsPEF effect when 2 mM of Ca^2+^ was substituted for 2 mM Ba^2+^ (Figure 2).

With 2 mM of extracellular Ca^2+^, a single 300-ns pulse elicited two distinct types of responses. At the low electric field strengths of 2.3–2.6 kV/cm, Calbryte emission increased immediately by 10–20% and remained near this level during 5 min of observation. At the electric field strength of 4.1 kV/cm or higher, the dye emission peaked to much higher levels, up to 2000%, in 10–20 s after the nsPEF, followed by a gradual decrease to 200–300% (Figure 2A,B). This highly nonlinear behavior results from the activation of CICR, when the entry of Ca^2+^ caused by the electroporation brings the cytosolic Ca^2+^ level to the CICR threshold of 200–300 nM [8,33]. CICR creates a positive feedback loop, which transiently raises the cytosolic Ca^2+^ to levels many times higher than with the electroporation alone, and activates active pumping of Ca^2+^ out of the cytosol [33]. These active processes obstruct the use of Ca^2+^ transients for quantitative measurements of electroporation and for monitoring of membrane repair after the nsPEF insult.

In contrast, Ba^2+^ entry caused predominately a smooth increase of the Calbryte signal (Figure 2C). The rate of the signal rise was the highest immediately after the nsPEF and then gradually decreased, presumably because of the membrane repair. The rate of the decrease was quantified by plotting the difference between the sequential time points versus time into the experiment [11] (Figure 2D). The decline of the signal with time was fitted with a double-exponential function, with fast and slow time constants of 6–11 s and 30–50 s, respectively. All curves came to zero within about 2 min after the electroporation, indicating that either the membrane was fully repaired, or the rate of Ba^2+^ entry became equal to the rate of its clearance from the cytosol. However, cytosolic Ba^2+^ did not appear to be sequestered by intracellular organelles [43]. The membrane repair explanation is further supported by the fact that both the double-exponential decline and the values of the time constants were similar to earlier findings with YO-PRO-1 dye [11], which binds to nucleic acids and does not get cleared from the cytosol (at least within 90 min of observation [26]).

### 2.3. Nominal Ca^2+^ and Intracellular Ca^2+^ Stores May Contribute to Ba^2+^ Entry Response to nsPEF

**Figure 3:**
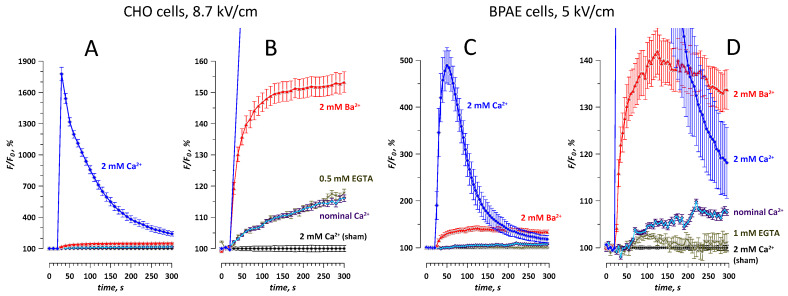
The effect of the ionic environment on the detection of membrane permeabilization in CHO and BPAE cells (**A**,**B** and **C**,**D**, respectively). Cells were loaded with Calbryte dye. Images were taken every 10 s (CHO) or every 5 s (BPAE). A single 300-ns pulse at the indicated intensity (kV/cm) was delivered at 27 s. Panels B and D show the same data as A and C, respectively, but on an expanded vertical scale. The labels identify whether the extracellular solution contained 2 mM Ca^2+^; or 2 mM of Ba^2+^; or none of them was added deliberately (“nominal Ca^2+^”); or the free Ca^2+^ level was further reduced with EGTA (“0.5 mM EGTA” and “1 mM EGTA”). Sham exposures were performed for all ionic conditions, with similar results; shown are the data for sham exposure in 2 mM Ca^2+^ only. Mean +/ s.e., 10–20 cells per each condition.

The nominal Ca^2+^ level in our extracellular solutions formulated without adding Ca^2+^ and without Ca^2+^ chelators was at 2–5 μM [26], which is still much higher than ~100 nM of the free cytosolic Ca^2+^ in quiescent cells. Therefore, the Calbryte traces in Figure 2C, which were presumed to reflect Ba^2+^ entry, could reflect some Ca^2+^ entry as well. The addition of Ca^2+^ chelators to Ba^2+^-containing solutions was avoided, because it would have reduced Ba^2+^ concentration as well and make data interpretation difficult. Instead, the contribution of the nominal Ca^2+^ was evaluated by comparing the traces of Calbryte fluorescence in CHO and BPAE cells exposed to nsPEF in different solutions (Figure 3). Both cell lines displayed similar patterns of the nsPEF effect. The response in the Ca^2+^-containing solution was by far the strongest (to 1900% in CHO cells and 700% in BPAE cells), due to its amplification by CICR. The same nsPEF exposure in the solution with nominal Ca^2+^ caused a weak (by up to 8–15%) but statistically significant (*p* < 0.01) response, without signs of CICR. The nsPEF effect in the solution with 2 mM Ba^2+^ was several times higher (to 140–150%, *p* < 0.01) and was clearly dominated by Ba^2+^ entry (which is reflected by the difference between “2 mM Ba^2+^” and “nominal Ca^2+^” traces).

**Figure 4:**
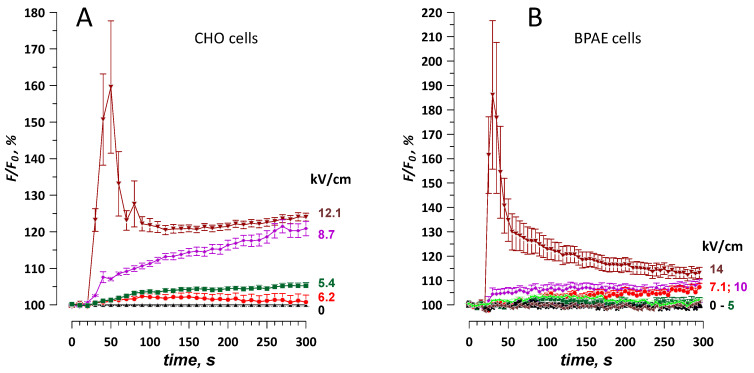
Mobilization of intracellular Ca^2+^ by intense nanosecond pulsed electric field (nsPEF) in CHO (**A**) and BPAE (**B**) cells. The extracellular solution did not contain any added Ca^2+^ or Ba^2+^ and was supplemented with 0.5 mM EGTA (CHO) or 1 mM EGTA (BPAE). The electric field strength (kV/cm) for a single 300-ns pulse is indicated in the labels. See Figure 3 and text for more details.

Adding a chelator (0.5 or 1 mM EGTA) to the nominal Ca^2+^ solution partially blocked the response to nsPEF (BPAE cells, Figure 3D) or did not change it (CHO cells, Figure 3B). The persistence of a significant response in the EGTA solutions, with the free extracellular Ca^2+^ reduced to less than 20 nM [44], suggests the mobilization of store Ca^2+^ by nsPEF [8,33]. Indeed, a single 300-ns pulse at the highest tested electric field strengths (>10 kV/cm) evoked a Ca^2+^ transient, which peaked at 160–190% in 10–20 s after the nsPEF, followed by a gradual decline to about 120% in 4–5 min (Figure 4). Weaker electric pulses (6–10 kV/cm) did not evoke the transient but caused a small gradual increase in fluorescence (to less than 110%, except for 120% for 8.7 kV/cm in CHO cells, Figure 4A).

To summarize, both the nominal and store Ca^2+^ could contribute to the fluorescent signal caused by Ba^2+^ entry. This contribution was negligibly small at low electric field strengths, but a transient Ca^2+^ peak at the highest electric field strengths could be comparable to Ba^2+^ response.

### 2.4. Different Time Courses of Ca^2+^ and Ba^2+^ Effects over an Extended Time Interval after nsPEF

In Figure 2, the fluorescence response to Ba^2+^ entry reached a stable value in about 2 min after nsPEF. In contrast, high Ca^2+^ transients continued to decline throughout the period of observation (300 s) without reaching a stable level that could be preferentially chosen to quantify the electroporation of the cell membrane. Therefore, we wanted to test if a stable level can be reached at longer time intervals, up to 30 min, after intense nsPEF treatments.

**Figure 5:**
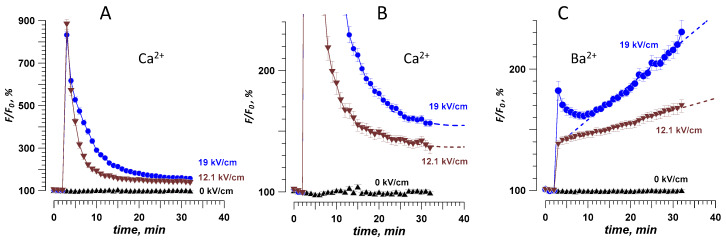
The different time course of fluorescence in Calbryte-loaded CHO cells during 30 min after nsPEF in the medium with 2 mM Ca^2+^ (**A**,**B**) or Ba^2+^ (**C**). Images were taken once a minute. A single 300-ns pulse at 0 (sham exposure), 12.1, or 19 kV/cm was delivered at 122 s. Panels A and B show the same data, with an expanded vertical scale in B. Dashed lines are the polynomial (**B**) and linear (**C**) fits through the last 10–15 data points. Mean ± s.e., *n* = 22–38.

The experiments established that Ca^2+^ transients after 12.1 and 19 kV/cm nsPEF continue to decline even in 30 min, albeit slowly (Figure 5A,B), and their projected time course beyond 30 min can be approximated with a second-degree polynomial fit. Such data could reflect either the slow reduction of Ca^2+^ influx due to the still ongoing membrane repair or the slow process of Ca^2+^ clearance towards the restoration of its basal level in already repaired cells.

Ba^2+^ responses showed a sharp initial increase followed by a steady and linear growth (Figure 5C). At 19 kV/cm, this growth was preceded by a transient peak, probably due to Ca^2+^ efflux from the intracellular stores (see Figure 4 and Section 2.3); a similar but smaller transient peak was expected at 12.1 kV/cm as well, but it might have been missed because of a long interval between image acquisitions (once a minute versus once every 10 s in Figure 4). The linear increase was 3-fold steeper at 19 kV/cm than at 12.1 kV/cm, reflecting faster Ba^2+^ influx through the more severely electroporated membrane. The linear increase also suggests failed membrane repair, in contrast to the membrane repair completed already in a couple of minutes after weaker nsPEF, up to 8.7 kV/cm (Figure 2C,D). The activation of cytosolic Ca^2+^ is required for many membrane repair processes [45,46,47,48], so its substitution with Ba^2+^ could have been an additional factor that prevented membrane repair after 12.1 and 19 kV/cm nsPEF.

### 2.5. The Dose-Effect and Sensitivity for Ca^2+^ and Ba^2+^ Detection Compared to the Uptake of Membrane-Impermeable Nucleic Stains

**Figure 6:**
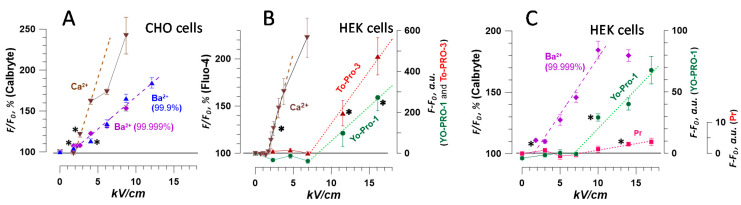
Dose-response and thresholds for the detection of membrane permeabilization by the entry of Ca^2+^ and Ba^2+^ ions or nucleic stains YO-PRO-1, TO-PRO-3, and propidium (Pr). The data were collected in CHO cells (**A**) and in two different sets of experiments in HEK cells (**B** and **C**). For Ca^2+^ and Ba^2+^ detection, cells were loaded with Calbryte dye (**A** and **C**) or with Fluo-4 (**B**). In all experiments, fluorescence images of cells were taken every 2–10 s for up to 4.5 or 9.5 min after nsPEF (traces not shown). Plotted are the values measured in the last image of each time series (except for Ca^2+^ data in panel B, which are the peak response values measured in 20–30 s after nsPEF). Note different units and baseline values for Ca^2+^ and Ba^2+^ detection (100%, Y-axes on the left) and for the detection of the nucleic stains (0 a.u., Y-axes on the right). The data points immediately above the baseline were fit with a linear function (dashed lines); the response thresholds were estimated by the intercept of the linear fit with the baseline. In panel A, the experiments with Ba^2+^ salts of different purity (99.9% and 99.999%) produced similar results and were pooled together for fitting. Asterisks designate the first measurement that was significantly above the baseline (*p* < 0.05 or better, one sample *t*-test). Mean +/− s.e., *n* = 14–40.

The electric field threshold was estimated by finding the intercept of a linear fit through data points with the minimal response to nsPEF with the baseline (100% for F/F_0_ or 0 a.u. for F-F_0_, Figure 6). The threshold for Ba^2+^ and Ca^2+^ entry after a single 300-ns pulse was 1.5–2 kV/cm for both CHO and HEK cells. This result matched earlier measurements in HEK cells, including cells transfected to express voltage-gated Ca^2+^ channels [24].

In CHO cells, the response was measured at the end of a 5-min recording, when it was less distorted by CICR (see Figure 2A,B). Nonetheless, the response amplitude at 6 kV/cm and above was lower than expected, probably because larger Ca^2+^ transients (amplified by CICR) caused faster Ca^2+^ clearance from the cytosol, yielding disproportionally low Ca^2+^ levels by the time CICR was over. In contrast, Ba^2+^ response increased almost linearly with the electric field (Figure 6A), and two independent series of experiments, performed 6 months apart, yielded very similar results. Of note, the purity of BaCl_2_ salt that was used to make the solution (99.9% and 99.999%) did not impact the results.

For HEK cells, we measured the peak amplitude of Ca^2+^ response [49], which increased almost linearly with the electric field (Figure 6B). In a parallel set of experiments, the uptake of YO-PRO-1 and TO-PRO-1 fluorescence stains was only observed at 11.5 kV/cm, with the threshold extrapolated to about 7 kV/cm. This same threshold was replicated in an independent series of experiments with YO-PRO-1 and Pr (Figure 6C). The uptake of Pr was barely above the noise level and became significantly different from zero only at 14 kV/cm (*p* < 0.05, one-sample t-test). Ba^2+^ response in HEK cells was already significant at 1.8 kV/cm (*p* < 0.05). Surprisingly, Ba^2+^ response did not increase at 3 kV/cm, which was first regarded as a “statistical glitch.” However, the same peculiarity was also seen in CHO cells (Figure 6A, data for 99.999% BaCl_2_): The amplitude of the response was the same for 1.8, 2.3, and 2.6 kV/cm exposures (*n* > 30 cells for each intensity, *p* < 0.0001 from sham exposure, two-tailed t-test). This result may be indicative of the involvement of at least two mechanisms of membrane permeabilization, differing in the dose dependence; however, more studies are needed to confirm it. Also, Ba^2+^ response did not further increase when the electric field was increased from 10 to 14 kV/cm, possibly because of Calbryte dye leakage from excessively damaged cells (Figure 6C).

**Figure 7:**
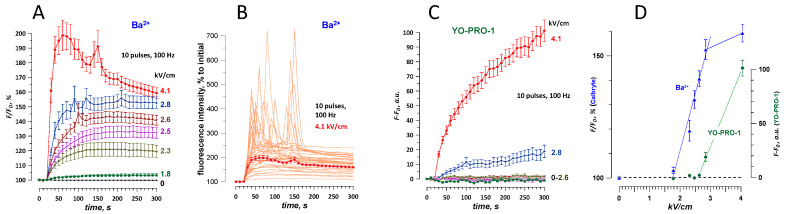
Permeabilization of CHO cells by a burst of 10, 300-ns pulses at 100 Hz. The membrane disruption was detected by a change in Calbryte fluorescence when cells were kept in a physiological solution with 2 mM Ba^2+^ (**A**,**B**) or by YO-PRO-1 fluorescence (**C**). Panel B shows sharp fluorescence peaks in individual cells exposed at 4.1 kV/cm and their average (+/− s.e.); the peaks were presumably caused by the mobilization of Ca^2+^ from the intracellular stores. Panel **D** shows the dose response for nsPEF effect traces in panels A and C, using the last data point in each time series. The electric field thresholds were estimated by linear fitting of the plotted values (see Figure 6 and text for more detail). Mean +/− s.e., *n* = 26–52.

The membrane permeabilization was expectedly more pronounced when applying a brief burst of 10, 300-ns pulses at 100 Hz (Figure 7). The threshold for Ba^2+^ response to such a burst was under 1.8 kV/cm, and for YO-PRO-1 uptake it decreased to about 2.5 kV/cm. Gradual increase of Calbryte fluorescence due to Ba^2+^ entry was “overlaid” by high-amplitude Ca^2+^ transients even at relatively low electric field strengths of 2.6–4.1 kV/cm, presumably because of the disruption of intracellular Ca^2+^ stores by nsPEF, which caused Ca^2+^ efflux and CICR (Figure 7A,B). All Ca^2+^ transients ended within 3 min after nsPEF and did not affect fluorescence readings at the end of the experiment (4.5 min after nsPEF, Figure 7D). However, the linear response range was rather narrow, and already at 4.1 kV/cm the signal was smaller than expected (Figure 7D), possibly because of the dye sequestration or leakage from severely damaged cells.

### 2.6. Fast Imaging of Ca^2+^ and Ba^2+^ Entry and the Asymmetry of nsPEF Effects

Studies with millisecond-range electropermeabilization pulses established preferential entry of Ca^2+^ from the anode-facing pole of the cell [50]. The authors suggested that the transient permeation structures formed on the anode- and cathode-facing cell poles behaved in different ways, but the underlying mechanisms have not been understood. For permeabilization by nsPEF, the admission of dyes like Pr and YO-PRO-1 is often localized and differs at the anode- and cathode-facing. However, no such difference could be observed for Tl^+^ or Ca^2+^ entry after nsPEF [28,51]. It remained to be seen if still faster image acquisition was needed to reveal polar uptake of these ions, or if nanopores formed diffusely over the plasma membrane surface, as suggested by the ‘‘supraelectroporation’’ model [9,18].

**Figure 8:**
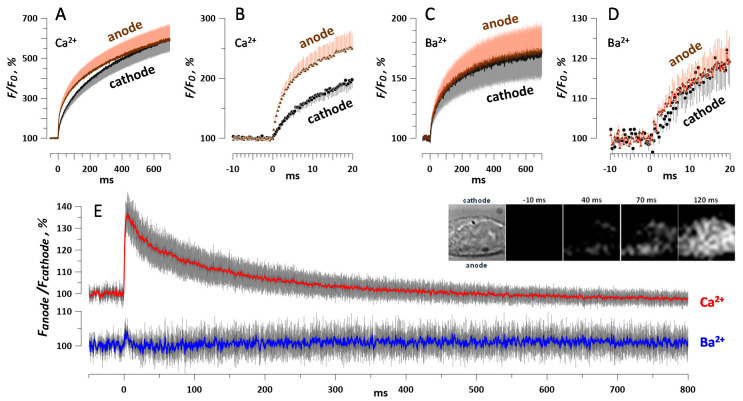
Polarity effect in the permeabilization of HEK cells by a single 600-ns pulse at 10 kV/cm. Cells were loaded with Calbryte dye and incubated in the physiological solution with 2 mM Ca^2+^ (**A**,**B**) or 2 mM Ba^2+^ (**C**,**D**). The acquisition rate was 3048 frames/s and the pulse was delivered at zero time point. Panels B and D show the same traces as A and C, respectively, at a higher time resolution. Panel E shows the difference in the fluorescence measured at the anode- and cathode-facing poles of each individual cell, averaged for all cells in the group. The inset illustrates preferential anodic entry of Ca^2+^ in a representative cell. The first image taken in the bright field shows the cell boundaries and the directions to the cathode and anode electrodes relative to the cell. The next images show the Calbryte fluorescence signal at indicated times before and after nsPEF. All images are 17 × 17 µm. Mean +/− s.e., for nine experiments with Ba^2+^ and 13 with Ca^2+^; error bars are plotted in one direction only, except for panel E. The difference between Ca^2+^ readings from the opposite poles (**E**) is significant at *p* < 0.01 (one-sample *t*-test).

Boosting the image acquisition rate to over 3000 frames/s revealed a transient difference in Ca^2+^ signal from the anodic and cathodic poles of the cell (Figure 8A,B,E). The emission increased at both poles already in the first image after a 600-ns, 10 kV/cm pulse. The increase at the anode-facing pole was initially about 1.4 times higher, but the difference gradually tapered out within the next 500 ms (Figure 8E).

Unexpectedly, this asymmetry of the response to nsPEF disappeared almost entirely when the external Ca^2+^ was substituted for Ba^2+^ (Figure 8C,D,E). The signal at the anode-facing pole was only 5–10% brighter instantly after the nsPEF, and just 10 ms later this difference was gone (Figure 8E). The striking difference between Ba^2+^ and Ca^2+^ responses suggests that nanopore formation by nsPEF was indeed very similar at both poles of the cell (as reported by Ba^2+^ entry), but Ca^2+^ response at the anodic pole was locally amplified and extended by Ca^2+^ store disruption, CICR, store-operated Ca^2+^ entry, or by some other process [38]. Alternatively, Ca^2+^ response at the cathodic pole could be attenuated by fast Ca^2+^ sequestration into the endoplasmic reticulum [38,52]. It is, however, difficult to explain why the active amplification or attenuation of Ca^2+^ responses would occur at just one but not the opposite pole of the cell. The identification of the exact mechanism involved could shed new light on the nanoporation phenomenon and its immediate consequences for cell function, but it was beyond the scope of this study. The fact that two similar approaches yielded qualitatively different results emphasizes the complexity of the nanoporation process and the necessity of combining different techniques for its analysis.

## 3. Discussion

We have introduced and characterized a new semi-quantitative method of analysis and monitoring of the permeabilized state of the cell membrane by the fluorescence detection of entry of Ba^2+^ ions. This method was compared to fluorescence detection of Ca^2+^ and of the uptake of membrane-impermeable nucleic acid stains YO-PRO-1, TO-PRO-1, and Pr. Different degrees of the cell membrane permeabilization in CHO, BPAE, and HEK cells were achieved by the electroporation with 300- or 600-ns electric pulses at various electric field strengths, from 1.8 to 19 kV/cm.

In contrast to Cd^2+^ and Zn^2+^ ions, Ba^2+^ did not penetrate into intact cells and did not change fluorescence in cells preloaded with Ca^2+^-sensitive fluorophores Calbryte or Fluo-8. A disruption of the cell membrane with a sufficiently strong nsPEF (over 1.5–2 kV/cm) could be detected within less than 1 ms by the fluorescence detection of Ba^2+^ or Ca^2+^ entry. This electric field strength matched earlier observations in diverse cell types, which consistently reported 1–2 kV/cm thresholds for a single pulse (200- to 600-ns duration) when the electroporation was detected by patch clamp, Tl^+^ uptake, or Ca^2+^ uptake [23,28,35,36,41,53]. However, the electric field threshold for membrane disruption detectable by the uptake of the nucleic stains was several times higher, above 7 kV/cm (Figure 6). This difference could be attributed to the larger molecular size of the stains compared to the metal cations, which can be admitted into the cell via smaller membrane pores [14,27,28,30]. Although dyes like YO-PRO-1 and Pr are commonly used for the detection and measurements of cell membrane disruption by nsPEF, such measurements are always limited to the larger subpopulation of pores and do not necessarily correlate with the transport through smaller pores [14,30]. For example, when 600- and 60-ns treatments were tuned to cause the same electrolyte and water uptake, the former caused 3-fold more Pr uptake than the latter [14]. This difference highlights the need to monitor the nanopore formation and resealing directly, by measuring the transmembrane transport of small solutes or water.

While Ba^2+^ and Ca^2+^ ions have approximately the same size and water mobility, the entry of Ca^2+^ into the cytosol triggers a myriad of active biological responses, which by themselves change the cytosolic Ca^2+^ level and may obscure the nanopore transport. The initial Ca^2+^ entry may be magnified by CICR disproportionally to the extent of the membrane damage, and the subsequent reduction of the cytosolic Ca^2+^ may reflect its active pumping out of the cell rather than the restoration of the membrane integrity. For example, CICR in CHO cells can increase the cytosolic Ca^2+^ level 10 times more than its increase solely due to Ca^2+^ entry through the electroporated membrane [33], so the inflow of Ca^2+^ can be estimated only in the presence of CICR inhibitors.

Within studied limits, Ba^2+^ was a more quantitative marker of the membrane electropermeabilization than Ca^2+^. It did not evoke CICR and was not appreciably cleared from the cytosol within 5 to 30 min of observation, which is consistent with prior reports that Ba^2+^ ions are at best only poorly pumped out by the plasma membrane calcium ATPase [54,55]. These features enabled monitoring of the membrane repair by Ba^2+^ entry (Figure 2D). However, most if not all known active membrane repair mechanisms in living cells are Ca^2+^-dependent [45,46,47,48], hence the substitution of the external Ca^2+^ for Ba^2+^ could inhibit the repair. Ba^2+^ may also inhibit K^+^ channels [56] and, by blocking the repolarizing action of K^+^ channels, facilitate membrane depolarization [40]. Ba^2+^ can stimulate the release of Ca^2+^ from intracellular sites, block Ca^2+^ extrusion from the cytosol [57,58], and modify the gating kinetics of Na^+^ channels [59].

Intense nsPEF treatments may trigger Ca^2+^ efflux from intracellular stores, causing a significant, albeit temporary, alteration of the fluorescence signal in a Ba^2+^-containing extracellular solution (Figure 5A and Figure 7A,B). Trace amounts of Ca^2+^ in the extracellular medium with Ba^2+^ had only a minor effect in our measurements but nonetheless are an additional factor to consider.

The possible entry of Ca^2+^ and Ba^2+^ through endogenous ion channels, either “spontaneous” or stimulated by nsPEF, can be another problem for the detection and monitoring of nanoelectroporation. Of note, the question of separation of flows through nanopores and through the endogenous ion channels is common for other methods of nanopore detection, whether using Tl^+^ entry [28], or cell swelling [14], or patch clamp [15,16,60,61]. This problem is eased by using cells like CHO and HEK, which have limited channelome and do not express voltage-gated Ca^2+^ channels; nonetheless, the possibility that other channels may actually form nanopores or contribute to the nanopore formation and stabilization cannot be excluded. When HEK cells were transfected to express CaV1.3 L-type voltage-gated calcium channels, Ca^2+^ entry in response to nsPEF expectedly increased, but by the end of the recording (70 s after nsPEF) the fluorescence signal had approximately the same amplitude in cells with and without Ca^2+^ channels [24]. The threshold for the fluorescence response did not change [24], possibly because the activation of Ca^2+^ channels was mediated by nanopore formation and the resulting loss of the resting membrane potential (instead of “direct” depolarization of the membrane by nsPEF, which was too brief for the activation of the channels). This indirect mechanism of activation of Ca^2+^ channels was also suspected in adrenal chromaffin cells exposed to 5-ns pulses, since the Ca^2+^ entry could be inhibited entirely with Ca^2+^ channel blockers, but was mediated by Na^+^ entry through nanopores [29]. In contrast, a presumed nanopore-mediated entry of Ca^2+^ evoked by 150–400-ns pulses could be isolated by applying a cocktail of inhibitors of voltage-gated Ca^2+^ channels [34].

Our experiments with the fast recording of Ba^2+^ and Ca^2+^ entry (Figure 8) not only reveal the asymmetry of the nsPEF effect on the entry of ions; our data suggest that some response specific for Ca^2+^ but not Ba^2+^ entry starts as early as in less than 1 ms after the stimulus, which is much faster than, for example, the generation of an action potential. This response would not be noticed if we only relied on Ca^2+^ detection of nanoporation, without comparing it to Ba^2+^ response. While no single method of nanopore detection and analysis is free from drawbacks and caveats, it is the combination of diverse methods that can identify nanopore properties and reveal how the membrane is repaired.

## 4. Materials and Methods

### 4.1. Cells and Media

Chinese hamster ovary cells (CHO-K1) and human epithelial kidney cells (HEK 293) were obtained from the American Type Culture Collection (ATCC, Manassas, VA, USA) and propagated as recommended by the supplier. Bovine pulmonary artery endothelial cells (BPAE) were a kind gift of Dr. J. Catravas (Center for Bioelectrics, ODU, Norfolk, VA, USA). These three cell lines were propagated, respectively, in Ham’s F-12 K medium (Atlanta Biologicals, Norcross, GA, USA); EMEM with 1.5 g/L sodium bicarbonate, non-essential amino acids, L-glutamine and sodium pyruvate (Mediatech Cellgro, Herndon, VA, USA); and a low-glucose DMEM with 2.5 µg/mL amphotericin B (Thermo Scientific, Waltham, MA, USA). The media were supplemented with 10% fetal bovine serum (Atlanta Biologicals, Norcross, GA, USA) and antibiotics (100 IU/mL penicillin and 0.1 mg/mL streptomycin, Gibco, Gaithersburg, MD, USA). Cells were grown in flasks at 37 °C, 5% CO2, and 1–2 days prior to experiments were passaged onto glass coverslips (BPAE and HEK) or into glass-bottomed 35-mm Petri dishes (MatTek, Ashland, MA, USA).

### 4.2. Solutions and Fluorescence Dyes

Experiments were performed in a physiological solution containing (in mM): 140 NaCl, 5.4 KCl, 1.5 MgCl_2_, 2 CaCl_2_, 10 glucose, and 10 HEPES (pH 7.3, 290–300 mOsm/kg). Modifications to this solution included: The omission of CaCl_2_; the omission of CaCl_2_ and the addition of 0.5 or 1 mM EGTA; and the substitution of CaCl_2_ with 2 mM of ZnCl_2_ or CdCl_2_, or with 2 or 5 mM of BaCl_2_. These modifications are indicated in the figures and respective sections of Results. Ba^2+^-containing solutions for most experiments were prepared with the ultrapure BaCl_2_ salt (99.999% trace metals basis). For several sets of experiments (Figure 1, Figure 5C, and Figure 7), the solutions were made with 99.9% BaCl_2_. Both salts were compared in Figure 6A and no difference in the nsPEF effect was observed. All chemicals were from Sigma-Aldrich (St. Louis, MO, USA).

Nucleic acid stains YO-PRO-1 and TO-PRO-1 (1 µM; Life Technologies, Grand Island, NY, USA), and Pr iodide (5 µg/mL; Sigma-Aldrich) were utilized to detect membrane permeabilization [11,13,20,21,26,30]. The dyes were added to the unmodified physiological solution for the entire duration of imaging experiments, without any pre-incubation. Our confocal microscope workstation (see below) enabled sequential excitation and imaging of two dyes in the same experiment, provided that the dyes had adequate spectral separation [28,30].

In separate experiments, we measured a change in the fluorescence of a Ca^2+^-sensitive indicator preloaded into cells. In most experiments, cells were loaded with 5 µM Calbryte™ 520 AM (AAT Bioquest, Inc., Sunnyvale, CA, USA) in the physiological solution, for 15–25 min at room temperature and protected from light. Multiple experiments, especially those in Figure 3, established that Calbryte dye is also sensitive to Ba^2+^ ions. As indicated in the Results section above, in isolated sets of experiments we also tested other Ca^2+^ indicators, namely Fluo-8 AM (AAT Bioquest) and Fluo-4 AM (ThermoFisher Scientific, Waltham, MA, USA); they were loaded into cells for 30 min at 10 or 5 μM, respectively. Cells were rinsed several times with the unmodified or modified physiological solution, and left in the same solution on the microscope stage for the duration of the experiments with nsPEF (up to about 60 min). In a single series of experiments without nsPEF exposure, the bath solution was changed during the time-lapse imaging, as described in Figure 1 and Section 2.1.

### 4.3. Cell Imaging and Fluorescence Measurements

Cell membrane permeabilization by nsPEF and recovery were monitored by time-lapse fluorescence microscopy at three different workstations based on IX71, IX81, and IX83 inverted microscopes (Olympus America, Center Valley, PA, USA).

The IX71-based workstation, equipped with an iXon Ultra 897 back-illuminated CCD Camera (Andor Technology, Belfast, UK), pE-340 Fura LED Fluorescence Light Source (CoolLED Andover, UK), and Solis interface (Andor), was used for ultrafast imaging in HEK cells (Section 2.6). A single cell was imaged with a PlanApo N 60×, N.A. 1.42 objective (Olympus). To achieve the high acquisition rate of 3048 frames/s (0.24-ms exposure time), we utilized a cropped sensor mode and limited the image to a single cell (64 × 64 pixels with 4 × 4 binning); the camera sensor outside this area was physically shielded with an Optomask (Andor). For data analysis, the regions of interest (ROI) were selected manually at the anode- and cathode-facing poles of the cell. The total recording time usually was limited to 1 s.

All other experiments in HEK cells and those in BPAE cells were carried out at the IX81-based workstation with an FV1000 confocal attachment and utilizing FluoView software (Olympus). All experiments in CHO cells were performed with the IX83-based system equipped with an Orca Flash 4.0 V.3 sCMOS camera (Hamamatsu, Bridgewater, NJ, USA), a 488-nm excitation laser (Coherent, Santa Clara, CA, USA), and utilizing MetaMorph v. 7.10.2.240 software (Molecular Devices, Foster City, CA, USA). A small group of cells in the center of the field of vision was imaged with a 40×, N.A. 0.95 air objective. The total recording time was varied from 100 s to over 30 min, with a 2- to 60-s interval between sequential images. For data analysis, ROI were manually drawn around the perimeter of each cell.

Each workstation was equipped with an nsPEF generator and delivery system (see below), which were triggered and synchronized with the time-lapse image acquisition by a TTL pulse protocol using a Digidata board (models 1322A, 1440A, and 1550B) and Clampex software (Molecular Devices). Imaging always started before the exposure to nsPEF. Fluorescence intensity within the defined ROI was quantified using the MetaMorph or ImageJ software [62]. For each ROI, the average intensity measured prior to nsPEF delivery was taken as the baseline (F_0_). For fluorescence dyes that were preloaded into cells (Fluo-4, Fluo-8, and Calbryte), the intensity measured in each frame after the exposure (F) was first corrected for the background (B), as measured in each frame, in a cell-free area of the image, and then normalized to the baseline as (100% × (F−B)/(F_0_−B_0_)). In some series of experiments, this formula was simplified to (100% × F/F_0_), either because the background intensity was negligible, or because the cell-free area was too small (in the cropped sensor recordings). For fluorescence dyes that were present in the extracellular solution during the experiment and entered the cell as a result of permeabilization by nsPEF (Pr, Yo-PRO-1, and TO-PRO-1), the entry was quantified in arbitrary units as (F-F_0_) [11,12,15,26,30].

### 4.4. Nanosecond Pulsed Electric Field Generation, Exposure, and Dosimetry

The concept of how nsPEF is delivered to individual selected cells or groups of cells on a microscope stage and the numerical simulation of the electric field distribution were reported by our group earlier [8,28,30,33,36,53] and described in detail recently [63]. General exposure procedures were the same at all three workstations, differing only in the details of which exact model of the manipulator, pulse generator, triggering device, and oscilloscope were used. In all experiments except the fast imaging series (Figure 8), we used 300-ns pulses from a MOSFET-based generator which was custom-built in house at ODU [64]. The pulse onset and duration were controlled by a model 577 digital delay generator (Berkley Nucleonics, San Rafael, CA, USA), which, in turn, was triggered by a TTL pulse from a Digidata device. For fast imaging experiments, we used a single 600-ns pulse from a generator custom built at Vilnius Gediminas Technical University in Vilnius, Lithuania [65].

A pair of tungsten rod electrodes (100 μm diameter, 140–250 μm gap) connected to a pulse generator were positioned within the microscope field of vision so that the selected cells were centered between their tips, and then were lifted precisely to 50 µm above the coverslip surface. The precise positioning was accomplished with a robotic manipulator (MP-225 or MPC-200, Sutter Instruments, Novato, CA, USA). The electric field at the cell location was calculated by 3D numerical simulations using a finite element analysis software COMSOL Multiphysics, release 5.0 (COMSOL Inc., Stockholm, Sweden) [63]. The exact shape and amplitude of nsPEF were monitored and measured using a TDS3052B or TDS7404 oscilloscope (Tektronix, Beaverton, OR, USA).

### 4.5. Experiment Protocols, Statistics, and Fluorescence Data Analysis

All protocols were designed to minimize potential biases and to ensure the accuracy and reproducibility of results. All series of experiments included a sham-exposed parallel control group, in which cells were subjected to all the same manipulations, but the nsPEF amplitude was set to zero. The nsPEF exposures at various electric field strengths and sham exposures were alternated in a random manner. In all experiments, each cell or group of cells was subjected to nsPEF exposure only once. With experiment protocols which lasted 5–15 min or less, we performed several exposures, at different electric field strengths, of spatially separated areas on the same coverslip (or in the same glass-bottomed Petri dish). For any type of nsPEF exposure, we performed from 6 to as many as 15 independent experiments, with a single cell (fast imaging) or a cluster of 4–6 cells per experiment. The number of cells used for statistical analysis for each type of treatment is indicated in figure captions.

The data from sham-exposed cell samples were used to determine any “baseline” fluorescence changes resulting from factors other than nsPEF. Such factors included dye bleaching and/or sequestration and removal from the cytosol (for dyes preloaded into cells) or the unforced uptake or “sticking” to the cell surface (for dyes present in the extracellular solution during experiments). nsPEF exposure data were corrected by subtracting the “baseline” fluorescence change as averaged from all sham-exposed samples in each series. The same correction was applied to the sham exposure data itself, which expectedly yielded a constant “100%” or a “0 a.u.” value; nonetheless, the corrected sham exposure data were included in graphs to show the error bars.

Data are presented in graphs as mean values ± s.e. for *n* cells; for clarity, the error bars are often plotted in one direction only. Student’s *t*-test with Dunnet’s correction when applicable [66,67] was employed to analyze the significance of differences; *p* < 0.05 (two-tailed) was considered statistically significant. Due to multiple statistical comparisons made between different groups and at multiple time points, we minimized the use of special symbols to mark the statistical significance in the graphs. Instead, the statistical significance can be estimated from the gap between the error bars of the compared groups: A gap exceeding the length of the error bar indicates a significant difference at *p* < 0.05 or better [68].

## Abbreviations

nsPEF	Nanosecond pulsed electric field
CICR	Calcium-induced calcium release
Pr	Propidium
